# Opinion Formation on the Internet: The Influence of Personality, Network Structure, and Content on Sharing Messages Online

**DOI:** 10.3389/frai.2020.00045

**Published:** 2020-07-02

**Authors:** Laura Burbach, Patrick Halbach, Martina Ziefle, André Calero Valdez

**Affiliations:** Human Computer Interaction Center, RWTH Aachen University, Aachen, Germany

**Keywords:** opinion formation, personality traits, message spread, social networks, network types, latent process model

## Abstract

Today the majority of people uses online social networks not only to stay in contact with friends, but also to find information about relevant topics, or to spread information. While a lot of research has been conducted into opinion formation, only little is known about which factors influence whether a user of online social networks disseminates information or not. To answer this question, we created an agent-based model and simulated message spreading in social networks using a latent-process model. In our model, we varied four different content types, six different network types, and we varied between a model that includes a personality model for its agents and one that did not. We found that the network type has only a weak influence on the distribution of content, whereas the message type has a clear influence on how many users receive a message. Using a personality model helped achieved more realistic outcomes.

## 1. Introduction

Social networks such as Facebook, Instagram and Twitter are now integrated into most people's everyday lives. The users of social networks no longer just use them to keep in touch with friends, but increasingly facilitate social networks to search for information. Users also form opinions based on the information and contributions available in social networks. While searching for information and integrating it into their opinion formation, users are no longer just passive recipients of information in online social networks, but are also actively spreading their own opinions (Hóllig and Hasebrink, 2016; Li et al., 2017; Frees and Koch, 2018). Thus, the dissemination of new information is increasing and a broader range of opinions is voiced (Bakshy et al., 2012).

Social networks play a powerful role and not only influence the formation of opinion of individuals, but can also play a decisive role in political situations and decisions (Guille et al., 2013). It has been shown, that social networks have a strong influence on political decisions. One example for this was the American presidential election in 2008, where many people perceived a strong influence of Twitter on the elections (Hughes and Palen, 2009; Shang, 2019).

While the amount of information users receive has changed through social networks, we also have to consider that information can now be personalized through the individual users' interaction with the network and its structure (DeVito, 2017). On the Internet, users can find almost any information they are looking for. However, the amount of information available on the Internet is now so large that users are no longer able to consume all the information. In addition, users also find contradicting information on the Internet. The increasing availability of information on the Internet has led to the development of recommendation systems (Adomavicius and Tuzhilin, 2005). Aiming to make it easier for users to select information, these systems analyse the information available, filter it according to specific criteria and provide users with recommendations tailored to their needs (Burke, 2002).

While this makes the search for information on the Internet and in social media easier for users at first, this selection of information can also have negative consequences. In the past, for example, the number of voices that view social networks as something negative has increased due to the fact that political opinions have been deliberately influenced and political results manipulated (Stark et al., 2017; Shang, 2019).

So far, research does not tell us how much opinion-forming processes actually take place in social networks and we can not predict those processes, let alone their consequences, yet. However, it is very important to consider the impact that the use of social networks has on user opinion formation.

Opinion forming and the processes that influence the formation of opinions have been studied since the 1960s. In the meantime, aspects that were of little or no importance before the rise of the Internet have become relevant for the research of the formation of opinion by individuals. First of all, in social networks every user can express his opinion and reach a large number of other users by just resharing or reposting (Cheng et al., 2014). By other users reposting the opinion of a user, long cascades are created and the opinion is disseminated (Cheng et al., 2014). Users share information targeting to convince other users of their opinion. Social media simplified that users can convey one's opinion to other individuals.

Still, users need to be connected with the users that they want to convince. Each user's network is individual and different from the network of other users, and they themselves can expand or shrink the network, which in turn can affect the accessibility of users.

Nevertheless, one aspect that needs to be considered is that users differ in their sharing habits. Lottridge and Bentley (2018) investigated the motivation to share contents and the frequency of sharing news on public, social and private platforms. They differentiate between different types of forwarding. Users can share messages with other users, they can share information in a personal message or share information on a social network or public a content publicly. In their research, they found that users have different intentions with different forms of sharing. Users share information publicly, primarily when they want to contribute an ideology. In contrast, they send private messages primarily to tell stories that correspond to their own interests or the context in which the user finds themselves. The first group shared news in all channels; they share both publicly, socially, and privately. In contrast, the second group does not share news at all. The last group shares messages only in private and social channel. Matching this, they also found that the group that shared the least posts had a negative attitude toward online discussion, whereas the group that shared the most posts had a neutral attitude toward online discussion (Lottridge and Bentley, 2018).

To understand how the dissemination of information and thus also the formation of opinion in social networks takes place, it is necessary to first consider what motivates users have to express their opinion in social media and which personality traits the users have that publish their opinion in social media. It is further relevant to look at different network structures, as they can also influence how content is spread and to whom—to friends or other users.

## 2. Related Work

In this study, we consider what influences how messages are spread in online social networks using an agent-based simulation. Therefore, we explain what is known in theory about the spread of information and the formation of opinions in this section. We further introduce the latent process model, on which we built our simulation and explain further aspects that are important for the agent-based simulation.

### 2.1. The Study of Complex Systems

The consideration of the spread of news in social networks is based on a complexity. We can also speak of a complex social system. In other words, a system consisting of several ontological levels. This system can be divided into its micro- and macro-level, which represent interacting subsystems (Conte et al., 2012). To understand complex social systems, it is not enough to look at the individual parts and understand them, but the overall system is more than the sum of all individual parts. If a system or a behavior cannot be described by the individual parts or subsystems alone, but in the overall system more becomes visible, one also speaks of *emergence* or emergent behavior.

A helpful way to understand this emergent behavior is to simulate the individual subsystems (Epstein, 2007). In this study, we also simulate the subsystems or processes of the spread of information in online social networks to become an understanding of the overall system. To do so, we use agent-based modeling, what is a well-suited method here (Epstein, 2007; Calero Valdez and Ziefle, 2018). One advantage of the system-theoretical approach with agent-based models is that we can use it to simulate how networks are created and information is disseminated or to simulate similar processes. Rational choice models often play a role in this type of modeling (Gilbert, 2008). We also developed such a model for this study. Agent-based models are not created aiming at an exact representation of the real world, but they try to represent individual behavior as realistically as possible and thus always simplify reality. The models also enable a qualitative observation of the behavior of the system. Evaluating such models is difficult and requires an independent replication of the model as well as a comparison with other models and a validation (Rouchier et al., 2008).

### 2.2. Information and Opinions

First, we must notice, that whether or not information is spread in a social network using the technological infrastructure, is independent from the spread of an opinion in the users minds. Therefore, it makes sense to model both sides of this process, first information dissemination and second opinion formation.

### 2.3. Spread of Information

Research speaks of information dissemination, information spread, or information diffusion when a person or a group of people sends information in a network (Li et al., 2017). Information dissemination has already been analyzed in many ways and it has been considered which aspects influence information processing as well as which information is processed how fast and in what manner (Christakis, 2007; Zhang and Wu, 2011). There are a number of dissemination models and other methods that are used to understand the diffusion phenomenon.

The spread of information in networks is similar to the spread of disease in contact networks, however, while the latter requires a face-to-face interaction—thus have relatively low limit on the edge-degree of nodes—the former can spread much faster due to the fact that online social networks allow for thousands of followers. When the president of the United States retweets a post from a user, several million other users are immediately exposed to this type of information.

When social networks and the structure of social networks are analyzed, it is also possible to examine the relationship between individual users and to identify patterns in user interactions (Wasserman and Faust, 1994). Some studies, for example, have been concerned with finding opinion leaders. Java et al. (2007) have shown how influential bloggers can be identified and Goyal et al. (2008) have identified opinion leaders in social networks through actions and interactions.

While much about the structure of networks on opinion formation has already been studied mathematically (Albi et al., 2016; Toscani et al., 2018), little is known about the psychological reasons for the spread of information in online social networks. For example, we do not know why the information in social networks flows in a certain direction. In addition, while we know that opinion leaders exist, we know little about how much influence they have on opinion formation and who are the most important users in disseminating information apart from the opinion leaders. Also only little is known about which factors influence the information diffusion process (Li et al., 2017).

#### 2.3.1. Diffusion Models

So far, diffusion models are used for many different purposes. A use case that is relevant for us is how messages are spread or how the spread of messages can be stopped (Guille et al., 2013). Following, we explain some basics of diffusion models. Basically, the *diffusion process* can be divided into two basic components. The first basic component of the process is a certain structure. The structure consists of a diffusion graph. The graph shows who influences whom. The second basic component is the temporal dynamics of the *diffusion process*. It describes how the diffusion rate develops. The diffusion rate means how many nodes take over the information over time. In the course of the *diffusion process*, a node can either be activated or not. An activated node has received the information and is trying to spread it. Within a network a successive activation of nodes takes place, which is called *diffusion process*. In models that consider the dissemination of information on social networks, users are usually influenced only by the people they are connected to. It is assumed that information is disseminated through information cascades (Guille et al., 2013).

### 2.4. Opinions and Attitudes

Since the spread of information does not equate the spread of opinions, we must understand how opinions are formed. Opinions are typically voiced—they are public. However, opinions may differ from the attitude of a person. The internal attitude may differ from the external opinion.

Moreover, the term attitude refers to various phenomena. There is no uniform understanding of the term attitude, let alone a uniform definition. There is also no agreement as to whether the terms opinion and attitude are synonymous or different. There is the opinion that both terms mean the same and are interchangeable, but also the opinion that they describe related processes, but refer to different aspects of these processes (Meinefeld, 1977; Oskamp and Schultz, 2005). However, there is agreement that attitude is a tendency to evaluate an object positively or negatively and to react to it if necessary. In this article, we are also guided by this notion of attitude, which also corresponds to the definition of Oskamp and Schultz.

We used the latent process model by DeFleur and Westie (1963) as the theoretical basis for the simulation. It explains the emergence of attitudes. According to this model, attitude is a theoretical construct, whose state should be considered as unknown. DeFleur and Westie see the attitude as a process variable within the opinion forming process. The opinion forming process is a preceding process to the reaction as a following process. The three processes form together the latent attitude. The latent attitude of a person is visible due to an observable reaction of the person. The reaction can be cognitive, a change in belief, affective, a change in emotion, or behavioral, a change in interaction.

For example, reading a post about the Iranian missile launch on American Forces in Iraq on January 8th 2020, claiming that no damage was done to American soldiers, could lead to several reactions. The reader could change their belief about the severity of the conflict situation between USA and Iran; they could perceive an emotional relief about the severity, yet cognitively perceive the threat as equally strong; or they could post contradictory or agreeing information online. In the latent process model, the affective and cognitive component are relatively independent of one another. The behavioral component is governed by both cognitive and affective processes.

#### 2.4.1. Three-Component Model of Attitude

Attitudes can consist of a cognitive, affective and behavioral component (Oskamp and Schultz, 2005). The cognitive component is a person's thought of an adjustment object. This component is also called conviction. While the cognitive component refers to what a person thinks, the affective component relates to what a person feels. More precisely, the component incorporates the feelings or emotions toward an object. Ultimately, the behavioral component holds the concrete intentions of a person and how the person actually behaves toward an object (Eagly and Chaiken, 1993; Oskamp and Schultz, 2005; Hartung, 2006). If the three different components are compared, the importance of the affective component in particular can be emphasized because emotions are motivating and make a person behave more strongly than cognition (Oskamp and Schultz, 2005).

#### 2.4.2. The Consistency Theorem

The processes described above are also referred to as the *three-component model* (McGuire, 1985; Eagly and Chaiken, 1993). In the past, however, some criticism has also been voiced about this established model. One of them stresses that it is not clear how the individual processes or components relate to each other. Thus, there is disagreement as to whether the three components actually say the same thing or whether, contrary to this opinion, they differ so much from each other that it is better to divide them into three separate units. In addition, there is the opinion that the attitude does not always consist of all three components (Oskamp and Schultz, 2005). Meinefeld (1977) and Oskamp and Schultz (2005) assume that the three components are just different names for the same thing are considered proponents of the *Consistency Theorem*. On the other hand, the proponents of the *Separate Entities Model* reject the Consistency Theorem and consider the three components as separate processes (Fishbein and Ajzen, 1975).

#### 2.4.3. The Latent-Process Model

The basis for the *latent process model* was the emergence of the critique of the *Consistency Theorem* and the *Separate Entities Model*. The three models are connected by the fact that they all try to explain how attitudes arise. While the *Consistency Theorem* assumes a consistency between attitude and behavior, DeFleur and Westie criticize exactly this assumption of consistency. In their opinion, an inner process takes place between the appearance of an external stimulus and a behavior. This inner process cannot be observed directly (through visible behavior), but the *Consistency Theorem* and the *Separate Entities Model* aim to explain this inner process.

On the contrary, DeFleur and Westie are of the opinion that the visible behavior is not the same as this inner process. They also assume that in addition to attitude other (social) factors influence behavior. In their opinion, the theoretical construct “attitude” is a link to describe the connection between object and behavior. The attitude itself must, however, be regarded as unknown. In the *latent process model* there is also an unobservable process of attitude formation which takes place before the inner process or attitude. Furthermore, there is a reaction that follows the attitude. In her opinion, a stimulus triggers cognitive and affective processes and also the process of behavioral intention. Then either individual processes or a combination of the processes form the latent attitude. This latent attitude becomes visible through a cognitive or affective reaction or behavior. In this model, the attitude can therefore be regarded as a probability conception and says how likely a person is to behave toward an object similar to how he has behaved in the past. As a result, in the model, attitude does not necessarily explain how a person behaves, but rather shows the regularity of certain behavior patterns. In addition to this advantage of the model, another advantage is that no relationship between the individual processes is assumed. In contrast, it is possible that only one process takes place, but also that two or all three processes take place (DeFleur and Westie, 1963; Oskamp and Schultz, 2005).

Other Researchers (e.g., Xiong and Liu, 2014) have investigated opinion formation using latent internal opinions. However, to our knowledge none have investigated the differences in processes underlying the disparity between opinion and behavior using a process model.

### 2.5. Social Networks and Their Users

To consider how messages are spread in a social network, not only the nature of the network must be considered, but also the personality of the users. It makes sense to model the users and their personality as realistically as possible to be able to make replicable statements. Some studies (Bachrach et al., 2012; Kosinski et al., 2013; Dong et al., 2014) in the past have already pointed out that the personality of users of online social networks is related to the characteristics of the respective network. Once the users have been created as realistically as possible, the simulation can start. This is followed by an arbitrary or fixed number of simulation steps. In the individual steps of the simulation, they interact with other users and with the environment in which the users live. Some parameters are set to determine how likely which stochastic processes occur (Serrano and Iglesias, 2016).

We consider personality traits of users of online social networks and how users behave in social networks as a basis for the most truthful possible design of agents in our model. For this study, we use the Big Five personality model to design our social network users, because it is the most established model to describe the personality of individuals. Following, we first describe the Big Five personality model and then how these personality traits are correlated with the use of or behavior in online social networks.

#### 2.5.1. Big Five Personality

There are many different models that try to describe the personality of individuals. If one wants to describe the personality of individuals, one inevitably comes across the Big Five personality trait model. It is a very established concept to describe different personalities (Costa and Mccrae, 1992). The personality of the individual is described in the model on the basis of five characteristics: *Openness to experience, conscientiousness, extraversion, agreeableness* and *neuroticism*. *Openness* means that a person has a lot of imagination and intellectual curiosity. *Conscientiousness* is understood to mean that a person is careful and well-organized. A person with a strong *extraversion* personality trait is sociable and tends to look for simulation. The personality trait *neuroticism* refers to negative emotions such as anxiety and depression and is defined as emotional instability. An individual, with a pronounced *agreeableness*, is very cooperative and has a lot of compassion for his other people (Power and Pluess, 2015).

Although the *Big Five factors* were initially designed as individual personality traits, some studies have shown that the traits are interrelated (Watson and Humrichouse, 2006; Grant and Langan-Fox, 2007). Power and Pluess investigated 5,011 European adults in their study and for the first time investigated the common heredity of the five *Big Five personality traits* with a GREML (Genomic-relatedness—Matrix Residual Maximum Likelihood) approach (Power and Pluess, 2015). They found that all the personality traits correlate with each other and also that all of them, except of *openness* correlate with gender.

Of course, there are other models that describe the personality of individuals, but in this study we focus on the model of the Big Five personality traits as the most established model to describe personality.

#### 2.5.2. Personality and Social Networks

In 2011, two studies by Gosling et al. looked at the use of Facebook and also measured how the *Big Five personality traits* of users related to their use. In the first study, the participants gave self-disclosure about their Facebook use. The study showed, that users with high *extraversion* values are connected to many Facebook friends. In addition, *extroverted* persons comment more frequently on contributions. While a high level of *conscientiousness* is associated with people spending less time on Facebook, more *open-minded* people tend to post more photos on Facebook compared to other users (Gosling et al., 2011).

In the second study, the Facebook profiles of the respondents were quantified in advance by “observers” and evaluated with regard to possible personality traits to reduce the effect of the self-report. While they did not find the results on *conscientiousness* in this study, they again found the same results on *openness* and *extraversion* (Gosling et al., 2011). Confirming the results of Gosling et al., other studies tried to even forecast the personality of users based on their facebook profiles (Golbeck et al., 2011)

Bachrach et al. conducted a study in 2012 with 180,000 participants and thus a significantly larger sample. They found similar results as in the previously described studies. For example, they found that more *open* people also publish and like posts more frequently and join Facebook groups more often. In addition, they found out that people who are more *conscientious* mark less post with like, but publish many photos. As with the studies described above, further studies showed, that extroverted individuals are associated with more Facebook friends, publish and like posts more frequently (Cullen and Morse, 2011; Bachrach et al., 2012; Cheevasuntorn et al., 2017).

### 2.6. Modeling a Social Network

In addition to the agents of a simulation, the environment must also be simulated. In the environment—the area in which the agents “live”—the agents interact with each other (Serrano and Iglesias, 2016). For modeling social networks in simulation environments the structure of the network has to be mirrored into an artificial environment. This can be done by either replicating a real social network or by referring to artificial network topologies that has similar characteristics to real social networks.

The generation of artificial networks has been investigated since the 1960s (Wasserman and Pattison, 1996). Usually these models are based on real social networks (Leskovec et al., 2009). One difficulty in depicting online social networks is that they are usually large and have both insecure structures and overlapping groups. However, this difficulty can be overcome with the help of agent-based modeling. With this method, networks with similar properties can be built generatively (Barrett et al., 2009; Pham et al., 2013). Agent-based models also enable to examine a large number of networks with similar characteristics and thus to simulate real network behavior.

An important basis to model message spreading is the structure of the network. The real social affiliation can also be seen in the structures of social online networks (Zheleva et al., 2009). Therefore, we also used different social network structures to connect our agents with each other in the environment, i.e., the social network.

### 2.7. Network Topologies

**Figure 1:**
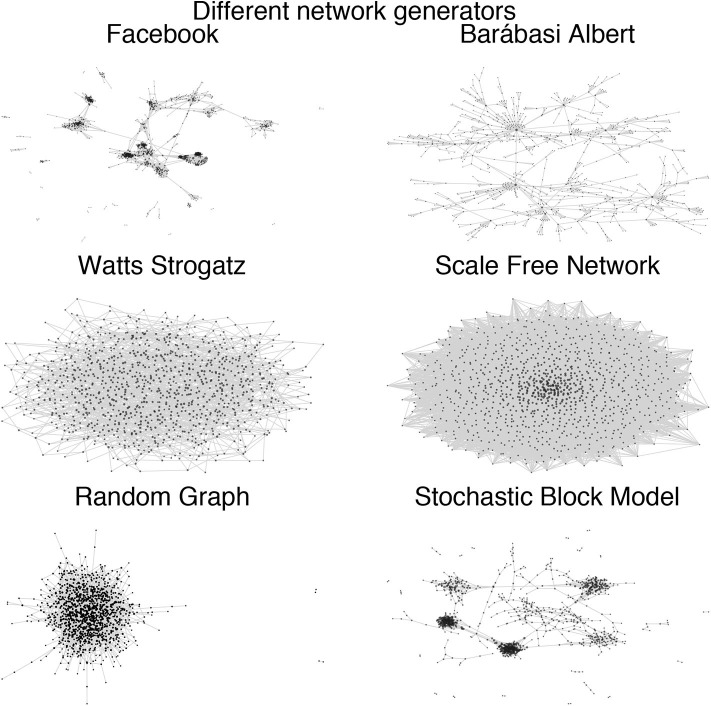
Different sample network topologies for 1,000 vertices: (top left) real Facebook data, (top right) Barábasi-Albert model, (bottom left) Watts Strogatz mode, (bottom right) Scale-free network. All Networks are displayed using a force-based layout.

Network topologies serve as a structural basis of social networks as they make it possible to understand the formation of node and link distribution and to describe effects that occur depending on the structure (see Figure 1). Network topologies can be classified into three types of networks: (1) Random Graph, (2) Scale-free, and (3) Small-World Networks that follow their own particularities (Albert and Barabási, 2002). In general it is important to know that several measurements can be provided to describe a network such as its centrality, cluster coefficient, and average path length. The clustering coefficient is an important value for examining the extent to which a network consists of local, strongly interconnected groups.

#### 2.7.1. Random Graph Networks

A random graph network is created by starting with a fixed set of vertices and adding edges between those vertices randomly. The most popular random graph model is the Erdős–Rényi model and actually combines two closely related models. The first model was proposed by Paul Erdős and Rényi and makes all graphs on fixed sets of vertices with a fixed number of edges equally likely. Contrasting, the second model proposed by Gilbert provides a fixed probability for each edge to exist or not, independently of the other edges (Erdős and Rényi, 1960).

#### 2.7.2. Small-World Networks

In a Small-World network model most nodes are indirectly adjacent to each other. This means that the average path length between the nodes is rather small, as every path between two nodes requires only a small number of hops. This implementation is needed to realize the small-world phenomenon that for example was investigated by Milgram and is connected to the idea that several networks follow the rule of “six degrees of separation” (Milgram, 1967). Small-World network properties appear in many real-world networks (Watts and Strogatz, 1998).

The Watts-Strogatz Model addresses this approach by extending the Erdős–Rényi model with an algorithm to create local clustering and triadic closure. It constructs a network with a regular ring lattice and rewires in a next step the vertices with a probability β for each edge while avoiding self-loops.This results in a graph with high local clustering and compared to a regular random network significantly reduced average path lengths through randomly rewired links. A minor drawback of this network is its weakness in producing realistic degree distributions as no hubs or scale-free distributions can be created.

#### 2.7.3. Scale-Free Networks

So-called scale free networks follow a power law distribution in terms of the network degree of their nodes. This means, that the degree of a node is proportionally related to its probability to get new connections. This results in an 80-20 distribution for the degree of nodes as 20% of the nodes are dominating 80% of the other nodes concerning their degree. This topology is predominant in social networks as for example the Erdős number shows: it describes, how close two scientists are in terms of collaboration, measured through their other collaborators in publications (Newman, 2001).

Such a network requires two main features considering its evolution process: it has to grow over time and the addition of nodes has to follow a preferential attachment strategy so that the probability for connecting a new node to the existing ones is higher for nodes who already have a high node degree than for those with low degree.

The Barábasi-Albert model describes a typical scale-free network topology. The algorithm serves for a power-law distribution of node degrees, resulting in a little amount of very well-connected hubs and a majority of nodes with only few connections to other nodes.

### 2.8. Stochastic Block Model

The Stochastic Block Model (Mossel et al., 2012) assumes that not all nodes stem from the same class. Speaking in network terms, users are from different countries, cities, social identity groups. The probability of nodes being attached to other nodes may now differ depending on class. Typically, nodes from the same class are highly interconnected, while only few connections are formed between classes. Using a stochastic block model community structures in the data can be explained, as found in real networks such as Facebook.

### 2.9. Facebook

Facebook is designed to allow users to maintain their connections, share content, and interact with content. However, Facebook was not intended to deliver news (Facebook, 2012). Regardless of what Facebook's original goal was and how the news feed was designed to serve that goal, the news feed is playing an increasingly important role in users' information flows. This is due to the fact that the user base has increased dramatically and users are increasingly integrating the news feed as part of their everyday lives (Duggan et al., 2014). Facebook is also becoming increasingly important as a source of news information. By 2014, 41 percent of Americans were already consuming news on Facebook (Matsa and Mitchell, 2014; DeVito, 2017).

Facebook in the role of the news source is responsible for some influential gatekeeping and agenda-setting functions that used to be more in the hands of human editors (McCombs and Shaw, 1972; DeVito, 2017). Here, Facebook intervenes primarily in the process of disseminating information by selecting which stories or topics are presented. Thus, it is no longer the editors who select the content to be presented, but the algorithms that are responsible for the selection of a story (boyd and Ellison, 2007; DeVito, 2017).

#### 2.9.1. Facebook Network

The Stanford Social Network Project provides a dataset for a Facebook Network consisting of 4,039 nodes and 88,234 edges. This data makes it possible to obtain a non-algorithmic, realistically grown network (McAuley and Leskovec, 2012). Stanford researches used this dataset to identify different types of social circles in online social networks such as friends or family members. We include this network dataset to compare the effects occurring in the other topologies to a realistic network.

### 2.10. Research Aim

With this study we aim to understand, what increases or decreases the spread of messages in social networks. To look at this question, we designed an agent-based model. Using this model, we focused on three different aspects and wanted to find out, how the three aspects influence the agents willingness to share a message. As a first factor, we considered four different types of content. Secondly, we considered five different network types and lastly we either considered the personality of the agents or did not.

#### 2.10.1. Other Types of Random Graphs

Many other types of random graphs, such as multi-type(Shang, 2016), bi-partite graphs, or stochastic block models exist. Some of these graphs might even be more suitable for the simulation of social networks. However, many of the properties about large scale components and their connectedness are similar to simple models anyways (Kang et al., 2015). As a first step, we choose to investigate single, unitype graph models in this paper.

## 3. Method

To study the effects of a dual-process model in different network settings we created an agent-based model to simulate message sending in networks using the Julia language (Bezanson et al., 2017). The simulation is written completely in Julia and available in a public GitHub Repository. Similarly, the data analysis is written in R using R Markdown and also openly available on GitHub.

### 3.1. Simulating Message Sending in Networks

To simulate how messages are sent in a network, we need to find ways to artificially instantiate the components that play a role in such a process. In our case we must simulate the individuals (the agents), the network, and the messages. We simplify our model, by assuming that only one message exists at a time. By running multiple simulations we can investigate the effect of different messages.

#### 3.1.1. The Message Model

We use a very simplified type of a message model. Messages contain two values, of which one describes the affective stimulus and the other marks the cognitive stimulus of a message. Both are drawn from four different options, which can be represented as a tuple [*val* = (affective, cognitive)]. We have chosen four message types that represent different the affective and cognitive values in different forms. We considered one message, that is mostly affective [*affective content*, *val* = (0.8, 0.2)], one message, that is mostly cognitive [*cognitive content*, *val* = (0.2, 0.8)], one content, that is both, affective and cognitive [*both*, *val* = (0.8, 0.8)] and lastly one weak content, that is rather neutral [*weak content*, *val* = (0.2, 0.2)].

#### 3.1.2. The Agent Model

The core idea of our study was to investigate the effect of the dual-process model in message sending. Thus our agents have virtual representations of the dual process model.

First, agents remember their affective and cognitive attitude toward a message. These attitudes are both drawn from the uniform distribution between 0 and 1 [*U*(0, 1)]. They are assumed to be statistically independent, which is reasonable as people may have different attitudes toward a subject on a cognitive or affective level.

Second, the behavior of the agents follows two individual thresholds. The noticing threshold (drawn from *U*(0, 1)) determines how much affective stimulation it requires to notice the content. By comparing the noticing threshold with the affective value of the message, it is determined whether the message is plainly ignored or evaluated further.

These variables were drawn from a uniform distribution to simplify the opinion space to a domain of [0;1]. In another experiment, we tested using normally distributed data *N*(0, 1), yielding very similar results. To match these variables to a domain of [0;1] we used a *arctanh*(*x*)-transformation.

Next, in case they noticed it, agents evaluate whether or not to forward a message. The posting threshold is compared with the full dual-process evaluation following a tripartite approach. The affective value of the message and the affective attitude both form the affective process variable by taking their mean. This entails that it requires both parts (attitude and message activation) for the process to play a role. Although, a weak activation can already trigger a relatively strong response (because we take the mean of both). The same is done for the cognitive process, i.e., take the mean of cognitive attitude and cognitive value of the message. Both processes are then combined using the geometric mean, making it necessary to have a strong activation on both processes to start the behavioral process. This last process is compared against the posting threshold. If the process value is higher than the threshold, the user forwards the message.

Simulating this process allows giving agents internal attributes and opinions that are not acted on unless a message activates them. But how to pick the thresholds?

We differentiate between two different agent models. The *random* agent, simply draws these thresholds from a uniform distribution [*U*(0, 1)]. This sets the expected threshold to 0.5 with strong variation in the sample [*E*(*SD*) ≈ 0.289].

The other agent we call *personality* agent, because we base this agent on a personality model. The underlying model is the Big Five personality model, from which we use three dimensions (Goldberg, 1990). Agents can vary with regard to extraversion, openness, and conscientiousness. There is evidence (see section 2) toward these measures that indicates that they influence behavior in social networks.

For example, higher extraversion of an individual increases its likelihood to have many connections on Facebook (Lönnqvist and Itkonen, 2014). Further, a higher openness makes it more likely for users to notice new content on social media (Alan and Kabadayı, 2016). Lastly, a higher conscientiousness decreases the likelihood to post content without thoughtful consideration (Gumelar et al., 2018/07). Thus, we derive the following: The *noticing threshold* in the *personality* agents is the mean of a random value from *U*(0, 1) and the inverse of the openness of the agent. This leads to a lower threshold for more open agents. The *posting threshold* is the mean of a random value from *U*(0, 1) and the conscientiousness of the agent. This increases the threshold for more conscientious users. The extraversion of an agent is later used in the network formation.

To create realistic personality values we draw these values from a multivariate normal distribution that is generated using the correlation (Table 1). To ensure that our values are in the domain of [0;1] we take the tangens hyperbolicus [which yields a domain of (−1;1)], add one, and divide by 2.

**Table 1:** Big Five Personality traits and relations to each other.

**Personality trait**	**1**	**2**	**3**	**4**
Extraversion	—			
Agreeableness	0.35^***^	—		
Conscientiousness	0.15^***^	0.27^***^	—	
Neuroticism	−0.24^***^	−0.05^***^	−0.20^***^	—
Openness	0.41^***^	0.22^***^	0.24^***^	−0.09^***^

Taken from Power and Pluess (2015). Statistically significant estimates are in bold. ^*^p < 0.05, ^**^ p < 0.01, and

*** *p < 0.001*.

This *personality* agent should behave more realistically than the completely *random* agent.

#### 3.1.3. Six Different Network Types

To measure the effect different random networks have on message spreading we generate six different network types. For this purpose we use the respective network generators supplied in the LightGraphs package (Bromberger et al., 2017) and the SNAP Dataset (Leskovec and Krevl, 2014). The scale free network uses the LightsGraphs implementation by Cho et al. (2009), therefore also referred to as “Cho.” The stochastic block model assumed 20 communities and randomly divides the agents to these communities. This is achieved by choosing 20 random numbers from a uniform distribution and dividing the by the sum. These numbers are then multiplied with the intended agent size. The weight matrix for the generator is randomly created by limiting the diagonal entries to values between 0.01 and 0.05 times the clustersize, and all other entries between 0.0001 and 0.01. For 60% of the non-diagonal entry we randomly also select 0, to achieve non-connected components. This achieves similar community structures as the facebook data used here as well.

**Table:** 

Overview of network properties by configuration				
In total 12.000 simulations were performed using 10 different settings.				
		Edges by network size (and standard error)		
Network type	# of simulations	1,000 agents	2,000 agents	4,039 agents
Personality based agents				
Barabasi Albert	1,000	999 ± 0.00	1,999 ± 0.00	4,038 ± 0.00
Facebook	1,000	5,409 ± 11.16	21,629 ± 26.24	88,234 ± 0.00
Random	1,000	1,996 ± 0.06	3,996 ± 0.06	8,074 ± 0.06
Stochastic block model	1,000	1,941 ± 11.89	3,911 ± 17.68	8,033 ± 25.52
Scale Free (Cho et al., 2009)	1,000	2,000 ± 0.00	4,000 ± 0.00	8,078 ± 0.00
Watts Strogatz	1,000	2,000 ± 0.00	4,000 ± 0.00	8,078 ± 0.00
Random agents				
Barabasi Albert	1,000	999 ± 0.00	1,999 ± 0.00	4,038 ± 0.00
Facebook	1,000	5,397 ±11.16	21,608 ±27.06	88,234 ± 0.00
Random	1,000	1,996 ± 0.06	3,996 ± 0.07	8,074 ± 0.06
Stochastic block model	1,000	1,934 ± 12.27	3,883 ± 0.07	8,059 ± 27.41
Scale Free (Cho et al., 2009)	1,000	2,000 ± 0.00	4,000 ± 0.00	8,078 ± 0.00
Watts Strogatz	1,000	2,000 ± 0.00	4,000 ± 0.00	8,078 ± 0.00

#### 3.1.4. Dual Process Model

We designed a latent dual process model to simulate opinion formation. The first process determines, whether an agent even perceives the contribution. This is determined by affective value of the message (*a*_message_). If it surpasses the noticing threshold (*t*_noticing_), the content is processed. In the *personality model*, the *openness* has an influence on this threshold—more open agents will have lower thresholds.

The second process simulates opinion formation based on the latent process model. Each message has two components an affective (*m*_affective_) and a cognitive value (*m*_affective_). The geometric mean of those values with the agents existing internal affective (*a*_affective_) and cognitive attitude (*a*_cognitive_) is then compared against a behavioral threshold. If the process evokes a stronger “reaction” than the threshold the user adapts the attitude and will now forward the message once to all neighbors. In the *personality model*, the *conscientiousness* of the agent determines this threshold—more conscientious agents will have higher thresholds.

**Algorithm 1:**
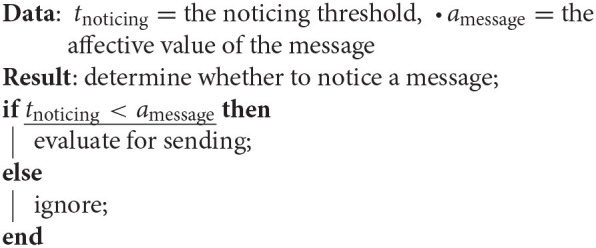
The noticing algorithm determines how a message gets noticed.

**Algorithm 2:**
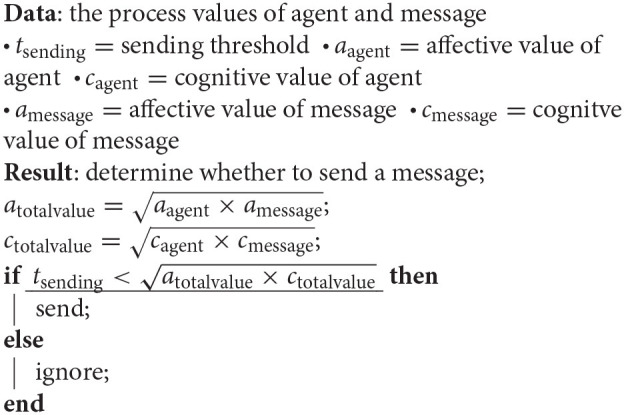
The sending algorithm determines whether or not an agents forwards the message.

### 3.2. The Simulation Procedure

Using the aforementioned model components we ran 30,000 different simulations. We varied the network size (1,000, 2,000, 4039 agents), the network generator (see above), and the agent type (personality or random). The node with highest vertex centrality was chosen as the starting node, to spread messages, simulating the behavior of an opinion leader that introduces novel content to their “sub-network”. We then ran the simulation until no more active senders were in the network. Agents that have sent the message, will not resend the message in later simulation steps. To reduce the impact of randomness we replicated each experiment 1,000 times using different random seeds. All experiments used a Mersenne Twister pseudo-random number generator. Initialization between different configurations of the experiments received the same random seed. Random seeds only varied for replications.

## 4. Results

Using the agent-based model, we analyzed whether three different initial settings lead to different outcomes. As initial configurations, we first used different content types (*affective content, cognitive content, both, weak content*). Secondly, we used different network types (*Facebook, Barábasi-Albert, Watts Strogatz, Scale Free Network, Random Network*).

**Figure 2:**
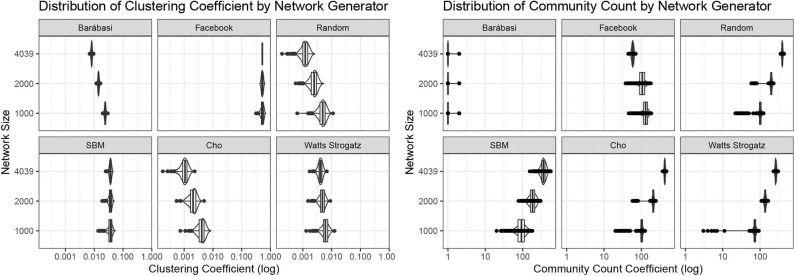
Clustering coefficients and community counts for all network generators.

We see that the network generators behave relatively stable regarding network size (see Figure 2). Both clustering coefficient and community count are stable. There are differences between the networks though. Real data from facebook shows the largest cluster coefficient in all settings. Only the stochastic block model seems to capture a high clustering coefficient equally well. The Barábasi Albert model only leads to one large community, as in our case we used a preferential attachment generator, starting from one node.

Thirdly, we compared whether the use of a *personality model* for the creation of the agents in our simulation leads to a different outcome than the simulation runs without the *personality model*. Using these three different initial settings, we found some interesting results, that we show following.

**Figure 3:**
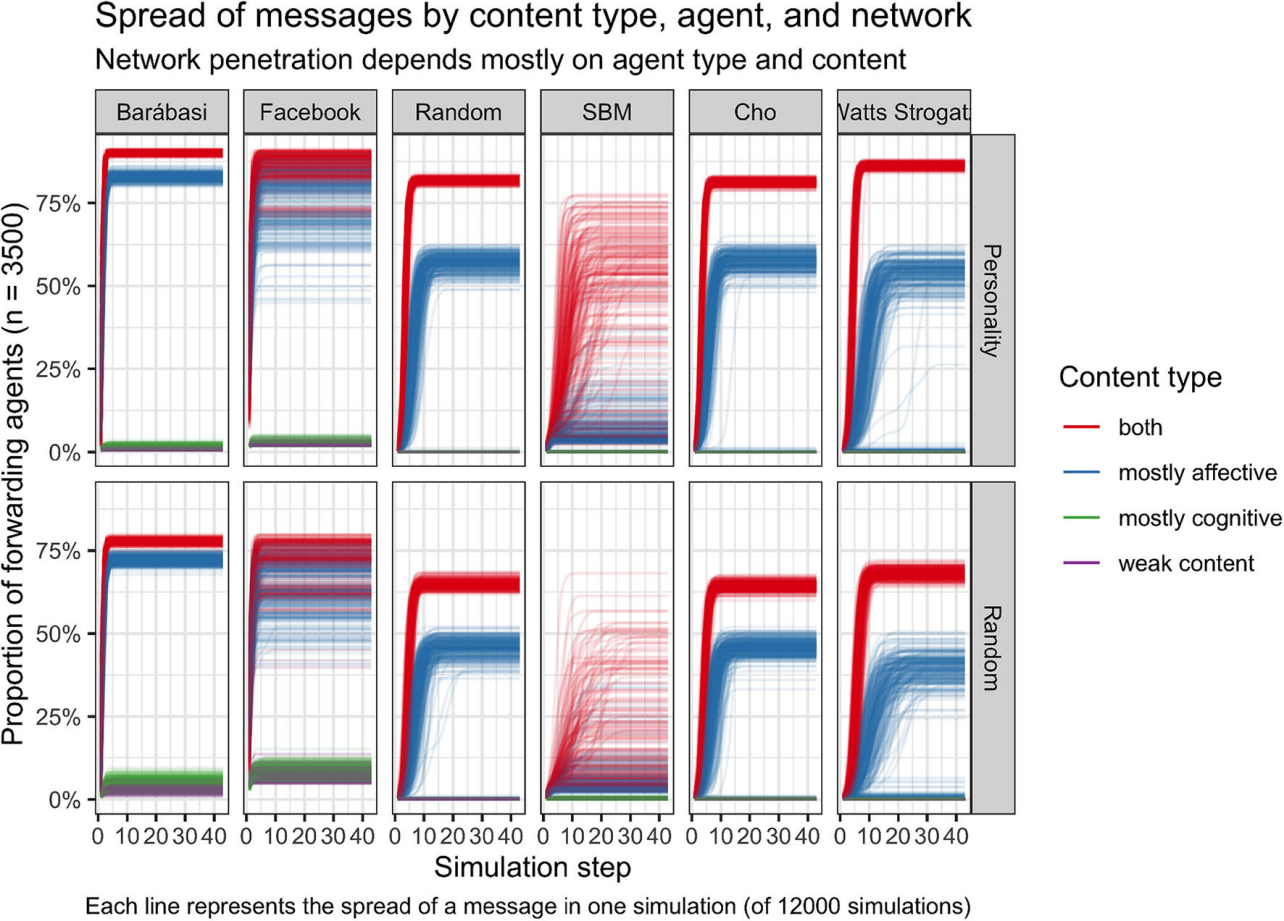
Differences in content type become apparent in the spread of information.

The results of our simulation runs are depicted in Figure 3. The figure shows for each simulation step how many of the agents who saw the message also forwarded it. The number of forwarding agents is also visible for the six different network types (horizontal); for the agents with and without the use of the *personality model* (vertical); and the different content types (color). The first aspect we look at in the following is when the number of forwarding agents or online social media users in our simulation is highest or lowest.

### 4.1. Highest and Lowest Proportion of Forwarding Agents

As can be seen in this figure, never all agents have seen and forwarded the message. This applies to all initial settings. The number of forwarding agents was highest in the simulation (which is shown above right), where the content is *both*, affective and cognitive, where the agents have an according to the Big five factors designed *personality*, and where the agents are located in the *Watts Strogatz* network. Using these initial settings, more than 75% of the agents did forward the seen content.

In contrast, the lowest number of forwarding agents occurred in the simulation (which is shown above left), where the content is *weak* or *mostly cognitive*, where the agents are designed according to the *personality model* and where the agents are located in a *Barábasi Albert* network. The agents stop forwarding the message at the latest at the fourth simulation step and until then almost no agent has forwarded the message.

So far we considered, when the proportion of forwarding agents is highest or lowest. Following, we look at the single factors that could have an influence on the proportion of forwarding agents, starting with the four different content types, that are highlighted in different colors in the figure.

### 4.2. Content Types

Comparing the four different contents, most agents see and forward the content, that is *both* affective and cognitive. In every network type apart of the *Barábasi Albert* network, where we used the *personality model* (upper row), did in the end more than 75% forward the seem content. In the *Barábasi Albert* network still more than 50% forwarded the content. Without the *personality model* (lower row), still more than 60% forwarded the content in the four other network types and in the *Barábasi Albert* network did more than 30% forward the content. The agents forward the *mostly affective* content the second most and significantly more frequently than the other two contents. The *weak content* as well as the *mostly cognitive content* are almost never forwarded at all. The agents also always stop forwarding the message before the eighth simulation step. Only with the *Facebook network* and without using the *personality model*, the *mostly cognitive content* is forwarded somewhat more frequently, but still forwarding does not exceed the eighth step of the simulation.

### 4.3. Network Type

After considering the influence of the content type, we now look at the different network types and how they influence the number of forwarding agents (horizontal). As can be seen in Figure 3, the proportion of forwarding agents differs only slightly between the *Random, Scale-Free*, and *Watts Strogatz* network. Hardly any difference can be seen between the red and blue lines of the forwarding agents located either in the *Random* or in the *Scale-Free* network. The lines also look very similar for the agents in the *Watts Strogatz* network. While in the *Random* and in the *Scale-Free* network many agents already forward the *both content*, the number of forwarding agents for the *Watts Strogatz* network is a bit higher. If we look at the *mostly affective content*, the number of forwarding agents in the *Watts Strogatz* network differs more for the individual simulations than for the two previously mentioned networks.

Slightly larger differences can be seen for the *Barábasi Albert* and the *Facebook* network. In the *Facebook* and the *Barábasi Albert* network, the number of agents that forward the *both content* and the *mostly affective content* is more similar. In the *Barábasi Albert* network, compared to all other network types, fewer agents forward the two most forwarded contents.

### 4.4. Personality Model

Lastly, we compare the message spread in our simulations based on whether the personality of our agents followed a *personality model* or was *randomly* generated. Figure 3 shows that the proportion of forwarding agents of the *mostly affective content* and the *affective and cognitive content* was always higher when they were equipped with a *personality model* in the *simulation*. The biggest deviation occurs when the agents are located in the *Barábasi Albert Network* or in the *Faceboook Network*. While in the *Barábasi Albert Network* around 20% of the agents forward the *affective and cognitive content*, when their personality is *randomly* generated, more than twice as many (around 44%) forward the *affective and cognitive content*, when their personality was designed through the *personality model*. Further, comparing the different ways of shaping the personality of the agents in the *Barábasi Albert Network*, the number of forwarding agents is almost identical for the other three contents.

In the *Faceboook Network*, the number of forwarding agents (with an intentionally created or random personality) is different for all contents. This type of network is also the only case where more agents with *random personality* forward the (*weak* and *mostly cognitive*) content than agents with an *intentionally created personality*. When the *personality model* was used, around 2% of the agents forwarded the *weak content* and around 3% forwarded the *mostly cognitive content*. Without the *personality model*, around 10% of the agents forwarded the *weak content* and around 11.5% of the agents forwarded the *mostly cognitive content*. In the end of the simulation runs, around 75% agents with a *randomly* generated personality forwarded the *affective and cognitive content*, with the *personality model*, the number of forwarding agents was around 87%. Around 62.5% of the agents with a *random* personality forwarded the *mostly affective* content and around 75% agents with an intentionally created personality forwarded the *mostly affective content*.

When the personality of the agents was *randomly* generated and the *Random, Scale-Free* and *Watts Strogatz* network was used, the number of forwarding agents (of the *cognitive and affective content*) remained below 75%. In contrast, the number was higher than 75% when we used the personality model. Likewise, the agents with an *intentionally created personality* forward the *mostly affective content* more often (around 44%), than the agents with *random personality* (around 25%). Regardless of how the personality of the agents is designed, no agent forwards the *mostly cognitive* and the *weak content*.

**Figure 4:**
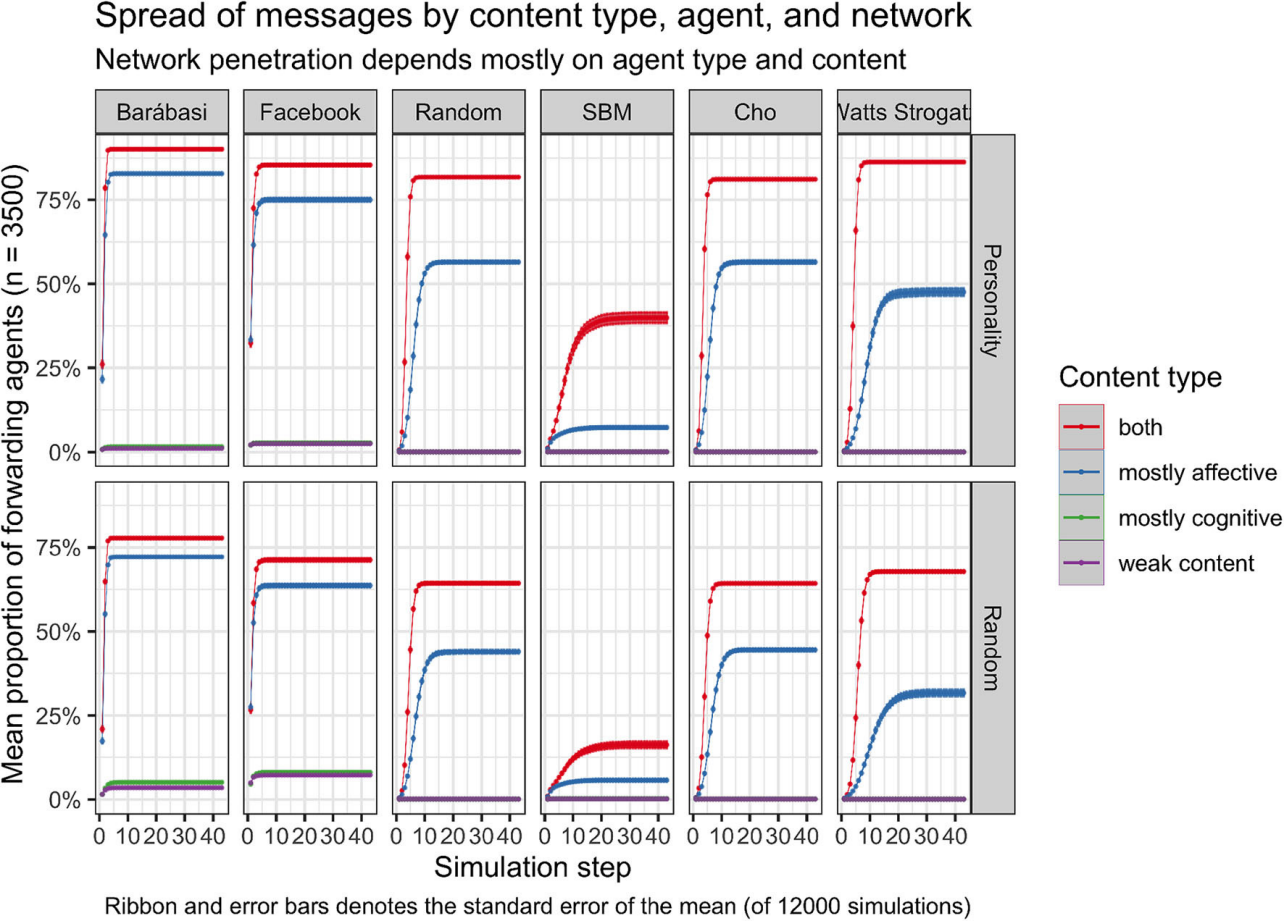
The deviation from the mean spread of message is low across all simulation.

Overall, Figure 3 shows that the proportion of forwarding agents mostly depends on the *content type* and if the agents have a *personality* designed according to the *personality model* or not. Even when looking at the standard error of the mean of proportions of agents that have seen or sent the message we see little deviation between the different graphs (see Figure 4) In contrast, the *network type* showed a lower influence except for *Barábasi Albert* and *Facebook* networks.

**Figure 5:**
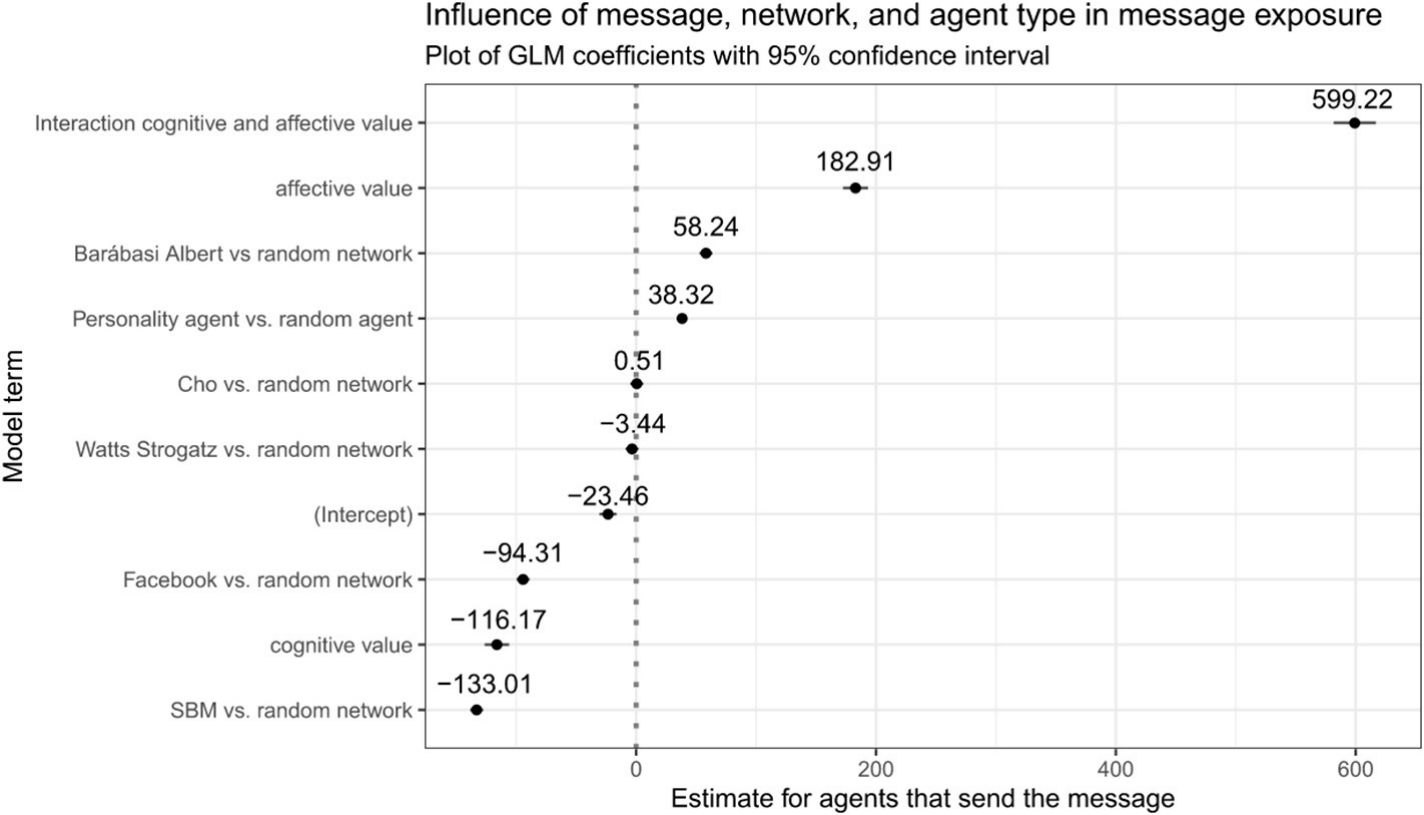
Plotting the coefficients of a general linear model to determine the influence of network and agent type.

Lastly, Figure 5 shows the results of a general linear model using agent type, network type and content type as predictors. As the predicted variable we used the number of agents that have sent the message until the last iteration step of each simulation. The model was significant with a null deviance of 473,939,679 on 11,999 degrees of freedom and a residual deviance of 93,791,997 on 11,990 degrees of freedom. The Akaike information criterion (AIC) for the final model was 141,644. This strengthens the importance of the message content in our model, but also highlights that the personality-based model contributed to modeling message spreading in our model. The only two factors not significant here were the network type being Cho or Watts Strogatz. As the null level of the network type the random graph was chosen. Our stochastic block model underestimated message spread compared against a real facebook model.

## 5. Discussion

The first result of our study was that the generators that we have used did not behave exactly as real world network data. The focus in our study was not to perfectly simulate the network, but investigate the effect a dual process model in such networks. We found that all synthetic networks shows lower clustering coefficients, which may be derived from the processes and parameters we used. The ratio of nodes to edges was by far higher for real world data then for our synthetic networks. Tweaking of these parameters in the future will provide additional insights regarding the network generators.

Interestingly, the qualitative behavior of all networks was similar, yet with different ultimate levels of spread. The stochastic block model, chose for its ability to reproduce community like data in facebook, shows by far lesser spread then the real facebook data. The scale-free network using the algorithm proposed by Cho et al. (2009) behaves rather non-intuitive. The implementation was used without verification from the “LightGraphs” package. Future studies should check for faults in this implementation as well.

Moreover, we did not look fully into different network sizes. The slices we have used have no inherent value except for their readability. This is important to understand as the behavior of the graph limits is very well-understood. Our models are still far from the graph limits, and thus future work should verify the impact of network size in this model as well as phase-transitions in the network (critical states).

The results of our study show that in no simulation all agents saw the content in the end. In no case did more than 80% of the agents see the content. This means that the forwarding of the content inevitably stopped at some point, but what caused the agents to stop forwarding the content? Why was it sent so far nevertheless?

The results of our study indicate that the personality of an online social network user and the type of content have the greatest influence on the spread of messages, according to our simulations. In contrast, it makes almost no difference whether the agents interact in different network types.

We assumed, that varying the network type would effect the distribution of content depending on how individuals are connected. However, we were not able to observe this in our study. Therefore, it may be less crucial to explore how users are connected in social networks and, in contrast, more important to consider how users perceive different types of content and whether and how this depends on their personality. Nevertheless, as we have shown in section 2, the personality also influences how many people a user is connected to in social networks. In addition, a user can only see a content if he is connected in some way to a person who is sending it, which means that the importance of different network types should not be neglected. This finding comes with a caveat. Network generators have additional parameters that can be changed to create different node to edge ratios. In our configuration we always had far fewer edges than the real-life representation of facebook. In this particular network, it is interesting to see that also mostly cognitive appealing content was spread further in the network. Boundaries between content types are not as clear in this case.

In a network that is based on the Barábasi Albert network topology, less users see and forward a message. A possible explanation for this can be seen in the structure of this network. Because of the preferential attachment of Barábasi Albert, few users exist who are connected to a lot of other users and if one of these well-connected users chooses to reject a message, this has a greater effect on the whole forwarding process than a rejection of a single user in the other network types. By providing better interconnectedness between all nodes, this effect is diminished in other network types as there is still a good chance that the other users will receive the message from another user with whom they are also connected.

Contradicting to Nekovee et al. (2007) who found that small-world networks facilitate a very high initial message spread compared to random networks, there seems to be no difference between random and small word networks in message spread (Nekovee et al., 2007). We also expected that the Watts-Strogatz performs better because of the “Strength of Weak Ties theory” of Granovetter (1973). But other studies also found advantages of network structures with high local clustering relying on the complexity of the adoption and forwarding process (Centola, 2010).

Confirmatory to the study of Xiong et al. the message spread of the scale-free networks and the Watts-Strogatz network as representative for small-world topologies is similar to each other (Xiong et al., 2015).

It has been shown that affective content is more likely to arouse readers' interest and also leads individuals to react to the content and forward it, for example (Oskamp and Schultz, 2005). Our results have also shown that the affective content is seen and shared much more frequently by social media users. Nevertheless, the combination of affective and cognitive content seems to increase the willingness to share a contribution the most.

As part of this study, we also designed the personality of the agents. In order to make the personality of the agents as realistic as possible and to resemble the personality of people or users of online social networks, we based our research on the results of the Big Five personality traits and how they relate to each other (see section 2.5.1). Thus, we designed the personality of the agents on the basis of the traits openness, conscientiousness and extraversion. Most interestingly, we see that including a personality model increases the reach of the message in all cases. This is partly due to the correlation of extraversion and openness in our model. More central nodes are more open and thus interact with more content. However, they are also more conscientious, but not sufficiently so to contain the spread of messages in a network. Integrating the personality perspective highlights the reach of both cognitively and affectively appealing messages. These show a very similar spread in many of the simulations.

By focusing this study on designing agent personality using three relevant features of the Big Five personality model, we omitted other features of the Big Five personality model and features of other personality models. This allowed us to design the relationships between the personality traits in a simple way and to design the personality of the agents in the model, which is always a simplification of the real world, in a sufficiently realistic way. Nevertheless, the personality of the agents can be designed more comprehensively. In the future, we would like to use the agreeableness and neuroticism of the Big Five personality model as well as other personality models to describe the personality of agents.

Further, we did not consider malicious individuals or *social bots* (Ferrara et al., 2016) in this simulation, although they have an influence on the spread of information in online social networks. Some studies (Bessi and Ferrara, 2016; Ferrara et al., 2016; Shao et al., 2018) showed, that social bots influence the public opinion by posting content and interacting with other social media users. Thus they behave like real social media users and are difficult to detect (Subrahmanian et al., 2016). Bots specifically send misinformation to users who are most likely to believe the information sent. This works well because people generally like to believe information that is popular or originates from their social environment (Jun et al., 2017). Using malicious agents/users or social bots in our simulation would have resulted in more spreaded weak content, what would have been interesting, but in this study (,as mentioned above,) we just concentrated on the influence of the three Big Five personality traits to keep the personality model relatively simple. In further studies we will extend the personality model not only by further personality traits, but also by different types of agents, such as malicious individuals, that try to manipulate the other agents in the simulation.

## 6. Conclusion and Outlook

With using an agent based model we found that the content type, the personality of a social media user and the type of network in which an individual is located have an influence on whether users see a contribution in the social network and whether they forward it. Overall, the willingness to forward a content depends more strongly on the content type and the personality of the agents than on the network type. This effect could even increase by using malicious agents or social bots. Still, there are special network types that have a great influence on how many users are reached by a content. The network type should therefore not be neglected in further research.

Since we saw that a network in which few users are connected to a lot of other users lead to a lower number of seeing and forwarding users, we want to consider network types with individual users connected to many other agents in the future. Here it could be particularly interesting to consider an agent of these well-connected agents as malicious.

Regarding the content type, a message, that combines affective and cognitive content increases the willingness to share the message the most. In contrast, social media users really do not want to share weak and mostly cognitive content no matter how the network is structured in our case. In the future it would also be interesting to take a closer look at the different forms of content. It is conceivable, for example, to compare different affective content to find out whether all affective content has a high probability to get forwarded or only content that appeals to certain emotions. It would also be interesting to look at different cognitively appealing contents or harmful content—e.g., click-bait, fake news, etc.—to find out what would make individuals forward that type of content more often than in our simulation or whether they actually never forward that content.

The integration of a personality model increases the willingness to forward content. At this point, however, we are not sure which other personality traits have the same effect and which personality traits could lead to the opposite effect and thus reduce the willingness to forward a contribution. It is conceivable, for example, that more conscientious persons would be even less likely to pass on harmful content. It is also conceivable that self-confident and extroverted social media as well as malicious users are more likely to forward content than people who fear negative feedback. In future, we will design the personality of our agents or social media users more comprehensively by including further personality traits other types of agents.

## Data Availability Statement

All datasets generated for this study are included in the article/supplementary material.

## Author Contributions

All authors listed have made a substantial, direct and intellectual contribution to the work, and approved it for publication.

## Conflict of Interest

The authors declare that the research was conducted in the absence of any commercial or financial relationships that could be construed as a potential conflict of interest.
